# Assay design for unambiguous identification and quantification of circulating pathogen-derived peptide biomarkers

**DOI:** 10.7150/thno.70373

**Published:** 2022-03-21

**Authors:** Qingbo Shu, Shan Liu, Tonino Alonzi, Sylvia M. LaCourse, Dhiraj Kumar Singh, Duran Bao, Dalton Wamalwa, Li Jiang, Christopher J. Lyon, Grace John-Stewart, Deepak Kaushal, Delia Goletti, Tony Hu

**Affiliations:** 1 Center for Cellular and Molecular Diagnostics, Department of Biochemistry and Molecular Biology, School of Medicine, Tulane University, New Orleans, Louisiana, USA.; 2 Sichuan Provincial Key Laboratory for Human Disease Gene Study, Department of Medical Genetics, Department of Laboratory medicine, Sichuan Academy of Medical Sciences & Sichuan Provincial People's Hospital, Chengdu, China.; 3 Translational Research Unit, National Institute for Infectious Diseases Lazzaro Spallanzani-IRCCS, Rome, Italy.; 4 Departments of Medicine, Division of Allergy and Infectious Diseases, and Global Health, University of Washington, Seattle, USA.; 5 Southwest National Primate Research Center, Texas Biomedical Research Institute, San Antonio, Texas, USA.; 6 Department of Pediatrics and Child Health, University of Nairobi, Nairobi, Kenya.

**Keywords:** MRM, peptide biomarker, immunoprecipitation, mass spectrometry, tuberculosis

## Abstract

**Rationale:** Circulating pathogen-derived proteins can serve as useful biomarkers for infections but may be detected with poor sensitivity and specificity by standard immunoassays due to masking effects and cross-reactivity. Mass spectrometry (MS)-read immunoassays for biomarker-derived peptides can resolve these issues, but lack standard workflows to select species-specific peptides with strong MS signal that are suitable for antibody generation.

**Methods:**Using a *Mycobacterium tuberculosis* (*Mtb*) protein as an example, candidate peptides were selected by length, species-specificity, MS intensity, and antigenicity score. MS data from spiked healthy serum was employed to define MS feature thresholds, including a novel measure of internal MS data correlation, to produce a peak detection algorithm.

**Results:** This algorithm performed better in rejecting false positive signal than each of its criteria, including those currently employed for this purpose. Analysis of an *Mtb* peptide biomarker (CFP-10pep) by this approach identified tuberculosis cases not detected by microbiologic assays, including extrapulmonary tuberculosis and tuberculosis cases in children infected with HIV-1. Circulating CFP-10pep levels measured in a non-human primate model of tuberculosis distinguished disease from asymptomatic infection and tended to correspond with *Mtb* granuloma size, suggesting that it could also serve as a surrogate marker for *Mtb* burden and possibly treatment response.

**Conclusions:** These biomarker selection and analysis approach appears to have strong potential utility for infectious disease diagnosis, including cryptic infections, and possibly to monitor changes in Mtb burden that may reflect disease progression or a response to treatment, which are critical needs for more effective disease control.

## Introduction

Closely related human pathogens that produce similar symptoms may cause different morbidity and mortality and require different treatments to effect cures. Such pathogens may be particularly difficult to distinguish if invasive procedures are required to isolate diagnostic specimens. Proteins secreted or shed by these pathogens, however, can serve as circulating diagnostic biomarkers, even for infections that occur at inaccessible or unknown sites. Such diagnostic proteins should ideally be expressed throughout infection and play essential roles in replication or virulence 1, but these properties can also limit their utility as specific biomarkers since proteins that play central roles in pathogen function tend to be highly conserved among related species 2, thus reducing the ability to develop species-specific immunoassays. Sequence differences in peptides derived from such proteins can, however, uniquely identify closely related pathogens 3, 4. Peptide biomarkers can thus serve as the basis for more precise diagnostics than traditional protein immunoassays but require a mass spectrometry (MS) readout to identify peptide sequence variations specific for individual pathogens. Carefully selected peptide biomarkers have the potential to distinguish a specific pathogen from other highly related members of an extended pathogen family, or uniquely identify multiple members with such families. Structural proteins of microbial cell walls or viral capsids often represent good candidates for biomarker peptides due to their relative abundance and shedding potential, but peptides derived from virulence factors may permit the discrimination of active versus latent infections to influence treatment decisions 5.

Assays that analyze serum-derived peptide biomarkers require affinity enrichment of either the target peptide from a digested sample or its native source protein for subsequent digestion, and each approach has its specific limitations. For example, affinity enrichment of the source protein from serum may be inhibited by competition due to non-specific interactions with abundant serum factors and/or specific interactions with targeted factors or high affinity host antibodies. Serum digestion can disrupt both these interactions but greatly increases the diversity of off-target molecules in the sample and thus requires increased specificity during the target enrichment and identification procedures. Several recent studies have focused on the latter biomarker detection approach.

Multiple reaction monitoring (MRM) MS analysis permits highly sensitive and specific detection of target peptides and their transition ions derived from biological samples, such as plasma or serum, that contain hundreds of proteins with broad and dynamic expression ranges 6, and subsequent quantification of these peptides. Commercial assays that employ peptide-specific antibodies, stable-isotope-labeled internal standard (IS) peptides, and MRM MS readouts have been developed to quantify host-derived proteins in human plasma 7. However, these assays typically detect and quantify peptides from host proteins, and thus do not specify conditions for differentiating highly related proteins, which can be critical for specific diagnosis of an infectious disease. Their targets can also circulate at much greater abundance than pathogen-derived factors that may serve as biomarkers of disease, particularly early in infection when pathogen burden may be low. This is reflected in the MS analysis criteria of these assays, which typically employs only three MRM transitions for target identification and quantification, and which potentially limits the ability to accurately detect low abundance signal among significant background noise that may be found in complex samples. Data analysis approaches such as AuDIT (automated detection of inaccurate and imprecise transitions) 8, 9 can analyze the relative intensity ratios of product ions produced by the target and IS peptides for significant differences to improve the specificity of target peptide detection. This analysis approach is used to produce the dot product and relative dot product (dotp and rdotp) similarity scores that the commonly used Skyline software package 10 employs to assess peak identity. However, despite the widespread usage of dotp and rdotp for peak identification, these scores can vary when adjusting peak boundaries and there are no established criteria for setting dotp and rdotp thresholds for peak identification.

We now describe an assay design workflow for identification of target peptides that differentiate closely related pathogen species and an MRM data analysis workflow for specific detection of low abundance pathogen-derived peptide biomarkers in complex samples. This MRM method uses an algorithm that defines acceptance thresholds for SNR, dotp, and rdotp values and define two correlation criteria that evaluate alignment of the detected transition ions of the biomarker peptide to improve rejection of spurious peaks from background noise. This study employs the *Mycobacterium tuberculosis* (*Mtb*) virulence factor CFP-10 to demonstrate these workflows and indicates that a peptide biomarker derived from this protein can detect CFP-10 concentrations as low as 0.5 pM in human serum, supporting the utility of this method.

## Methods

### Clinical Cohorts and Samples

Clinical research studies described individually below were performed following the STROBE-statement guidelines for observational studies 11, 12 and in accordance with The Code of Ethics of the World Medical Association (Declaration of Helsinki) 13. Plasma samples analyzed for the Italian adult TB cohort were collected in BD Vacutainer lithium heparin tubes (Cat. No. 367874, BD, Italy) and stored at -80°C until use. Serum samples analyzed for the Dominican Republic and Kenya pediatric TB cohorts were collected, coagulated, and centrifuged to separate serum in BD Vacutainer red-top tubes (3 mL, Cat. No. 366668, BD, USA or Kenya) using a standard clinical serum collection protocol, after which serum was transferred in microcentrifuge tube aliquots and stored at ‐80°C until use. All TB suspects enrolled in each study were evaluated using all microbiologic and clinical results obtained through the duration of their study participation, and all patients diagnosed with TB were diagnosed based on data collected prior to their initial analyzed plasma or serum sample.

### Multi-center Adult TB cohort

Adult plasma samples used to validate CFP10pep detection criteria were drawn from cryopreserved samples of an adult cohort containing 125 TB cases and 12 healthy controls (**Table 1;** see Supplemental methods for diagnosis criteria), who were consented and enrolled at multiple international sites (Morocco, Eritrea, Ethiopia, Italy, Romania, Sri Lanka, Kenya, Guinea, Brazil, Nigeria, Ukraine, Afghanistan, Poland, Swiss, Philippines, Senegal, Peru, Somalia, India, China, Nepal, Moldova) in a study was approved by the Ethical Committee of L. Spallanzani National Institute of Infectious Diseases (INMI) in Rome (approval numbers 34/2010 and 72/2015).

### Dominican Republic pediatric TB cohort

Serum samples used to evaluate IP-MS diagnostic performance in a pediatric TB cohort without significant HIV involvement were collected from 31 suspected TB cases enrolled in a prospective pediatric TB study performed in the Dominican Republic. This cohort contained 17 children (aged <18 years) diagnosed with TB (8 confirmed TB and 9 unconfirmed TB cases) and 14 classified as Non-TB cases who did not meet the criteria for TB diagnosis (unlikely TB diagnoses), as determined using NIH pediatric TB diagnosis criteria 14. All study participants, or their legal guardians, provided consent for study participation prior to enrollment, using an IRB protocol approved by the Ethics Committee of the Universidad Dominicana O&M School of Medicine.

### Kenya PUSH pediatric TB cohort

Serum samples used to evaluate IP-MS diagnostic performance in children living with HIV were obtained from cryopreserved serum of 19 children enrolled in the Pediatric Urgent Start of HAART (PUSH) cohort, a randomized controlled trial (NCT02063880) evaluating whether urgent (<48 hours) vs. post-stabilization (7-14 days) antiretroviral therapy (ART) improved survival in hospitalized HIV-infected children <12 years old in Kenya 15. This cohort contained 11 children diagnosed with TB (5 confirmed TB and 6 unconfirmed TB cases) and 8 classified as Non-TB cases who did not meet the criteria for TB diagnosis (unlikely TB diagnoses), as determined using NIH pediatric TB diagnosis criteria 14. All study participants, or their legal guardians, provided consent for study participation prior to enrollment, using an IRB protocol approved by the Kenyatta National Hospital and University of Nairobi Ethics Research Committee, and the University of Washington Institutional Review Board.

### Non-human primate (NHP) TB infection and sample collection

NHP plasma and bronchoalveolar lavage (BAL) fluid samples analyzed in this study were archived samples obtained from NHPs infected with *Mtb* in previous reported studies, and were repurposed for validation of the MRM assay in this NHP disease model 16-21. Briefly, specific-pathogen-free, retrovirus-free, mycobacteria-naive, adult rhesus macaques were assigned to three groups: *Mtb* naïve control group (n=4); a TB infection group (n=4) subjected to a low-dose aerosol exposure (~10 CFU of *Mtb* CDC1551) resulting in positive TSTs by 1 month post-exposure but no TB symptoms during the study (~22 weeks); a TB group (n=5) subjected to a high dose aerosol exposure (~200 CFU of *Mtb* CDC1551) that resulted in active TB characterized by weight loss, pyrexia, elevated serum CRP levels, elevated chest radiograph scores, detectable CFUs in BAL fluid, higher lung bacterial burden and associated lung pathology at study endpoints, where lung tissue was randomly sampled by blinded pathologists using a grid approach 22, 23.

### LC-MS/MS analysis of recombinant CFP-10 protein

Recombinant CFP-10 (Cat. No. 105-20, ImmunoDX) was dissolved in 100 µL of 50 mM ammonium bicarbonate, mixed with 0.5 µg sequencing grade modified trypsin (Cat. No. V5111, Promega), and incubated at 37 °C for 16 hrs, then a 5 μL aliquot (~0.25 µg) was analyzed by a QExactive HF-X mass spectrometer (Thermo Fisher Scientific) coupled with an UltiMate 3000 ultrahigh-pressure liquid chromatography (UHPLC) system. Samples were loaded on an Acclaim PepMap100 C18 trap column (300 μm ID × 5 mm, 5 μm, Thermo Fisher Scientific; Cat. # 160454), and separated on a PepMap C18 analytical column (75 μm ID×15 cm, 3 μm, Thermo Fisher Scientific, Cat. # 164568) using a 300 nL/min gradient generated by mixing buffer A (0.1% formic acid in water) and buffer B (0.1% formic acid, 99.9% acetonitrile) as follows: 5 min wash with 5% buffer B, 17 min 5-38% buffer B gradient, 2 min 38-95% buffer B gradient, 10 min wash with 95% buffer B, 0.1 min 95-5% buffer B gradient, and 0.9 min 5% buffer B wash.

### Prediction of Peptide Antigenicity

Three bioinformatic tools were used to generate peptide antigenicity scores for all peptides evaluated as potential targets for capture antibodies: 1) the Antigen Profiler Peptide Tool (Thermo, https://www.thermofisher.com/us/en/home/life-science/antibodies/custom-antibodies/custom-antibody-production/antigen-profiler-antigen-preparation.html), 2) the OptimumAntigen Design Tool (https://www.genscript.com/antigen-design.html), and 3) the BepiPred-2.0 linear epitope prediction tool (http://tools.iedb.org/bcell/), using the default cut-off scores of each tool (2.7, 0.6, and 0.5, respectively).

### Peptide-specific antibodies

Rabbit polyclonal and mouse monoclonal antibodies were raised against indicated *Mtb*-specific CFP-10 peptide conjugated to keyhole limpet hemocyanin (GenScript), purified from immune serum or culture supernatant by peptide affinity or protein An affinity (>99% pure by SDS-PAGE analysis), resuspended in PBS, quantified by A280 using a NanoDrop spectrophotometer, and then analyzed by indirect ELISA. For rabbit antibodies used to evaluate peptide antigenicity, wells on 96-well microtiter plates were incubated with 100 µL CFP-10 peptide 1 (1.56-25 ng/mL in PBS, pH 7.4, two-fold serial dilution) or peptide 2 (15.63-1,000 ng/mL, two-fold serial dilution) for 4°C for 16 hrs, PBS washed, blocked at 37°C for 1 hr with 200 µL blocking buffer (PBS, 5% BSA, 0.05% Tween-20), then incubated at 4°C for 16 hr with 100 µL antibody (1 µg/mL) in blocking buffer. For the mouse monoclonal antibody used in the final assay, wells on 96-well microtiter plates were incubated with 100 µL CFP-10pep (1 µg/mL in PBS, pH 7.4) for 4°C for 16 hrs, PBS washed, blocked at 37°C for 1 hr with 200 µL blocking buffer (PBS, 1% BSA, 0.05% Tween-20), then incubated at 4°C for 16 hr with 100 µL antibody (1.95-1,000 ng/mL) produced by two-fold serial dilution of a polyclonal immune serum in blocking buffer. Mouse and rabbit antibody ELISA wells were then washed four times with PBST, and respectively incubated for 0.5 hr at 37°C with 100 µL Peroxidase-AffiniPure Goat Anti-Mouse IgG or Peroxidase AffiniPure Goat Anti-Rabbit IgG (RRID: AB_2338513 and 111-035-144, Jackson ImmunoResearch Labs) diluted in 1:5,000 in blocking buffer, washed four times with PBST, incubated for 20 min at 25°C with 100 µL TMB Reagent (GenScript), then mixed with 100 µL of stop solution (1 M HCl), then for absorbance at 450 nm was read using a microplate reader. The limit of detection for each sample was the highest dilution with a signal-to-blank values ≥ 2.1, where the blank was the average value of two replicate wells that did not have primary antibody added to them. The immunoprecipitation yield for the mouse monoclonal antibody was evaluated by as the ratio MS peak areas produced by peptide recovered after immunoprecipitation versus the input amount when 2 picomole of IS was spiked into a 100 µL trypsin digest of healthy human serum.

### Serum and plasma digestion

Human and NHP serum or plasma aliquots (200 µL) were split into 100 µL aliquots that were each mixed with 900 µL denaturing buffer (PBS pH 7.4, 0.4% SDS, 0.2% triton X-100), incubated at 100°C for 5 min, and transferred to a 25°C water bath for 5 min. Samples were then spiked with 10 µL of unbuffered 1 M Tris base solution (Bio-Rad) to achieve pH 8.5, mixed with 10 µg sequencing grade modified trypsin, incubated at 37°C for 16 hours with rotary mixing, and then adjusted to pH 7 by addition of 10 µL of 10% (v/v) trifluoracetic acid (TFA). Samples analyzed for target peptide quantification were then spiked with 5 µL of a 500 nM solution of IS peptide containing a C-terminal stable-isotope-labeled arginine (^13^C_6_^15^N_4_) (GenScript). Standard curves for CFP-10pep quantification were generated by spiking commercial serum from a healthy donor (Cat. No. H4522-20ML, Sigma-Aldrich) with serial dilutions of recombinant CFP-10 (0.5 - 10 pM) prior to sample digestion.

### IP-MS analysis of target peptides

Protein G-coupled Dynabead aliquots (3 mg; ThermoFisher Scientific) were washed with 200 µL of PBS pH 7.4 with 0.2% Tween-20 (PBST-2), incubated with 50 µg antibody in 400 µL PBST-2, washed twice with 200 µL PBST-2, and resuspended in 1 mL PBST-2. Conjugated Dynabeads were then incubated with digested plasma or serum (0.15 mg beads/sample) for 1 hr at 25°C with rotary mixing, washed twice with 100 µL of PBS, once with 100 µL of LC grade water, then incubated for 30 min at 25°C with 100 µL of 1% (v/v) formic acid solution prior to magnetic separation to isolate eluted peptide supernatants, which were washed on StageTips before MS analysis (see Supporting Information). MRM analysis was performed on an Ultimate 3000 UHPLC system with an Altis triple quadrupole mass spectrometer (Thermo Scientific), using the same column and LC conditions used for LC-MS/MS analysis recombinant CFP-10 analysis. Both Q1 and Q3 resolution (full width at half maximum, FWHM) were set to 1.2. These analyses used a 1.5 mTorr collision gas pressure, a 2.2 kV spray voltage, a 275°C capillary temperature, a 40 V collision energy setting and a 40 msec dwell time for each transition, with a 131 V RF lens setting.

MS/MS spectra were searched against a customized database containing CFP-10 and 236 common contaminant proteins using Peaks Studio software (version Xplus, Bioinformatics Solutions, Canada) and Maxquant (version 1.6.10.43), using default settings for tryptic digestion, and precursor and product mass tolerances. Cysteine carbamidomethylation was defined as a stable modification, and methionine oxidation, and protein N-term acetylation as dynamic modifications. Identified CFP-10 peptide spectra were imported into Skyline (version 20.1.0.76) to build its spectral library. MRM data were imported into FreeStyle (version 1.5, Thermo Fisher Scientific), and signal-to-noise ratios (SNRs) in extracted ion chromatograms were calculated using the Genesis peak detection algorithm, which was set to detect all peaks with SNRs ≥ 2. Similarity scores (dotp and rdotp) and peak area of targeted peptides were exported from Skyline.

MRM raw data was converted into mz.ML format using ProteoWizard 24 (version 3.0.19282), selecting the SRM spectra option. The extracted ion chromatogram window was determined to be 41 scans wide using spike-in data (**Figure S1**). Data collected in this window was analyzed for correlations among its intensity matrix in Matlab (R2020a, Mathworks), and exported as correlation coefficient matrixes or cross-correlation plots and used to determine correlated ion pair numbers, negatively correlated ion pair percentages (%ρ_ip_^-^), and sum of significantly correlated ion pair coefficients (∑_s_ρ_ip_^±^). Batch processing of MRM data extraction and analysis of transition time series correlations used a python package using code SciPy-1.6.3 (https://docs.scipy.org/doc/scipy/reference/generated/scipy.stats.kendalltau.html). Modified code for MRM-MS data analysis is available at https://github.com/FanLab2019/MS_correlation.

### Training and testing datasets for CFP-10pep peak criteria

Specific thresholds were peak acceptance (dotp, rdotp, SNR) and noise filtering features (%ρ_ip_^-^ and ∑_ss_ρ_ip_^±^) were established in a training set that used MRM data from digested and immunoprecipitated triplicate samples of commercial healthy donor plasma (Sigma) spiked with or without 0.5 pM recombinant CFP-10 protein. SNR, dotp and rdotp values corresponding to positive signal were required to exceed acceptance thresholds defined as the mean-3×SD value of the matching parameter in the LOD sample (0.5 pM CFP-10), while %ρ_ip_^-^ and ∑_ss_ρ_ip_^±^ values were required to fall below and above the lower and upper 95% confidence interval of these values determined in the blank samples (0 pM CFP-10). The ability of these criteria to accurately reject non-specific signal in the CFP-10pep detection window were evaluated individually and in aggregate in a testing data set that used plasma from 20 healthy donor adults (12 adults without evidence of TB in the Italian cohort and 8 commercial samples obtained from healthy donors in the US).

### Statistical analysis

Unpaired parametric t-test and Mann-Whitney tests were performed using GraphPad Prism (version 9.2.0) to analyze pairwise comparisons, where test selection was determined by the normal distribution and variance of the data. Differences were considered significant at p-values of ≤0.05.

## Results

### Selection of a peptide biomarker and its MRM transitions

*Mtb* and its virulence factor CFP-10 were selected for this study since *Mtb* belongs to a large family of mycobacteria, many of which express CFP-10 orthologs; *Mtb* is the primary cause of tuberculosis (TB), a leading cause of death from infectious disease, which can be difficult to diagnose by current methods in several patient populations at high risk for morbidity and mortality 5; CFP-10 is actively secreted by virulent *Mtb* strains and plays a key role *Mtb* survival and virulence 25; and since circulating CFP-10 can serve as direct evidence of an active *Mtb* infection and diagnose multiple TB manifestations 26, but its levels in blood can span a wide dynamic range and thus require highly sensitive and specific detection methods 27.

Since immunoaffinity enrichment approaches require development of high affinity and specificity antibodies, early identification of appropriate peptide targets is essential for rapid development of effective assays. However, rules employed for selection of peptide biomarkers derived from host proteins are not directly applicable to pathogen-derived proteins. For example, several non-tuberculous mycobacteria (NTM) express CFP-10 orthologs 28, including two of six NTM responsible for >80% of human mycobacterial respiratory isolates 29, restricting the peptides available as biomarker targets. We thus employed multiple criteria to identify CFP-10-derived peptides that could serve as biomarkers TB disease. Candidate peptide biomarkers were required to 1) contain ≥ 7 amino acids, 2) exhibit specificity/selectivity for *Mtb*, 3) produce strong MS signal, and 4) exhibit robust potential antigenicity as an affinity enrichment target. Sequence analysis of *Mtb* CFP-10 identified seven tryptic peptides ≥ 7 amino acids, five of which had variable sequence conservation with the two most common NTM respiratory pathogens expressing CFP-10 orthologs and strong MS signal intensities (**Figure S2**). However, only one peptide (CFP-10pep) passed the acceptance thresholds of three antigenicity prediction algorithms and was subsequently captured with high affinity by antibodies raised against it. A mouse CFP10pep-specific monoclonal antibody subsequently exhibited 31.9% recovery of a CFP-10pep IS peptide spiked into a healthy human serum digest at low concentration (2 pmol; **Dataset S1**).

Next, seven CFP-10pep y-ions (y5-y11) were selected as MRM transitions due to their high MS signal intensities in trypsin digests of recombinant CFP-10 protein and low number of predicted interferences (**Figure S3** and **Dataset S2**). Co-elution of these y-ions produced a characteristic peak at the retention time (RT) defined by the stable-isotope-labeled CFP-10pep (heavy) IS peptide, where the target window was defined by the full width at base of the CFP-1pep measure over 41 repeat scans of a sample (**Figure S4** and** S5**).

### Evaluation of CFP-10pep MRM MS peak features

**Figure 1:**
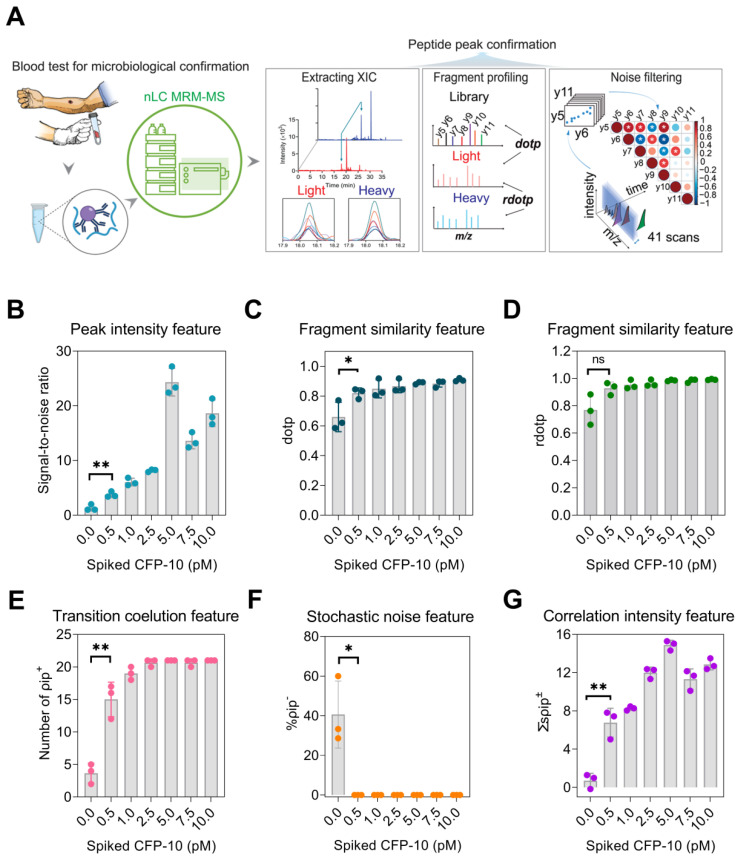
** MRM-MS assay approach to detect peptides from circulating pathogen-derived biomarkers for species-specific diagnosis.** (A) Schematic of the assay approach: a target peptide is enriched from a serum or plasma digest spiked with a stable-isotope-labeled internal standard (IS) peptide by immunoprecipitation, and is then analyzed by LC MRM-MS. The extracted ion chromatograms of the labeled IS peptide is aligned with that of the endogenous peptide (blue and red lines) to identify the retention time window (light blue arrows) of the target peak and extract each of its MRM transitions as deconvoluted peaks (colored lines). Selection criteria (SNR, dotp, rdotp; and ion pair correlation values) for peak identification are calculated from values obtained from low concentration control or blank samples, respectively, to distinguish the weak target signal from background noise. (B) SNR, (C) dotp, (D) rdotp, and the (E) number of positively correlated ion pairs (ρ_ip_^+^), (F) sum of significant ion pair correlation coefficients (∑ρ_ip_^±^), and (G) percentage of negatively correlated ion pairs (%ρ_ip_^-^) exhibit variable change with CFP-10 concentration. Data are shown as mean ± SD (n = 3) indicating significant (*p < 0.05 and **p < 0.01) and non-significant differences (n.s.) versus the blank (0 pM) sample by unpaired parametric t-test.

To distinguish CFP-10pep signal from noise, feature information was extracted from peaks and transition ions eluting within the CFP-10pep RT window and evaluated their SNR, dot product (dotp) and relative dot product (rdotp) (**Figure 1A**). SNR was employed to evaluate the probability that detected peaks represented signal versus electronic noise, while dotp and rdotp were used to evaluate how well transition ions peak areas detected within this window matched those of a reference library sample or the IS values of the same sample, respectively. SNR, dotp and rdotp values for peaks detected in the CFP-10pep RT window increased with CFP-10 concentration (**Figure 1B-D**), but only SNR and dotp distinguished peaks detected in the 0.5 pM CFP-10 sample from noise in the blank sample. SNR, dotp and rdotp values are often used to improve the accuracy of target peak identifications 30, but analyze signal from all candidate ions detected within a target window, regardless of their relative elution profile alignments. Since cross-correlation analysis of these alignments has been reported reduce false classification, we evaluated the classification performance of three correlation parameters in negative control samples: the number of positively correlated ion pair (ρ_ip_^+^), the negatively correlated ion pair percentage (%ρ_ip_^-^) and the sum of the significantly correlated ion pair coefficients (∑_s_ρ_ip_^±^) in each sample. This analysis found that ρ_ip_^+^ events were enriched in samples spiked with 0.5 pM CFP-10 versus PBS (mean 15 vs. 4, **Figure 1E** and **Table S1**), and ρ_ip_^-^ events were detected only in blank serum samples (**Figure 1F; Figures S4** and **S5**) where they accounted for ~40% of the significant correlations. All three parameters (ρ_ip_^+^, %ρ_ip_^-^, and ∑_s_ρ_ip_^±^) distinguished CFP-10pep peaks in 0.5 pM CFP-10 spike-in samples from noise in control serum. Notably, ρ_ip_^+^ events and ∑_s_ρ_ip_^±^ increased with CFP-10 concentration (**Figure 1E** and **1G**).

**Figure 2:**
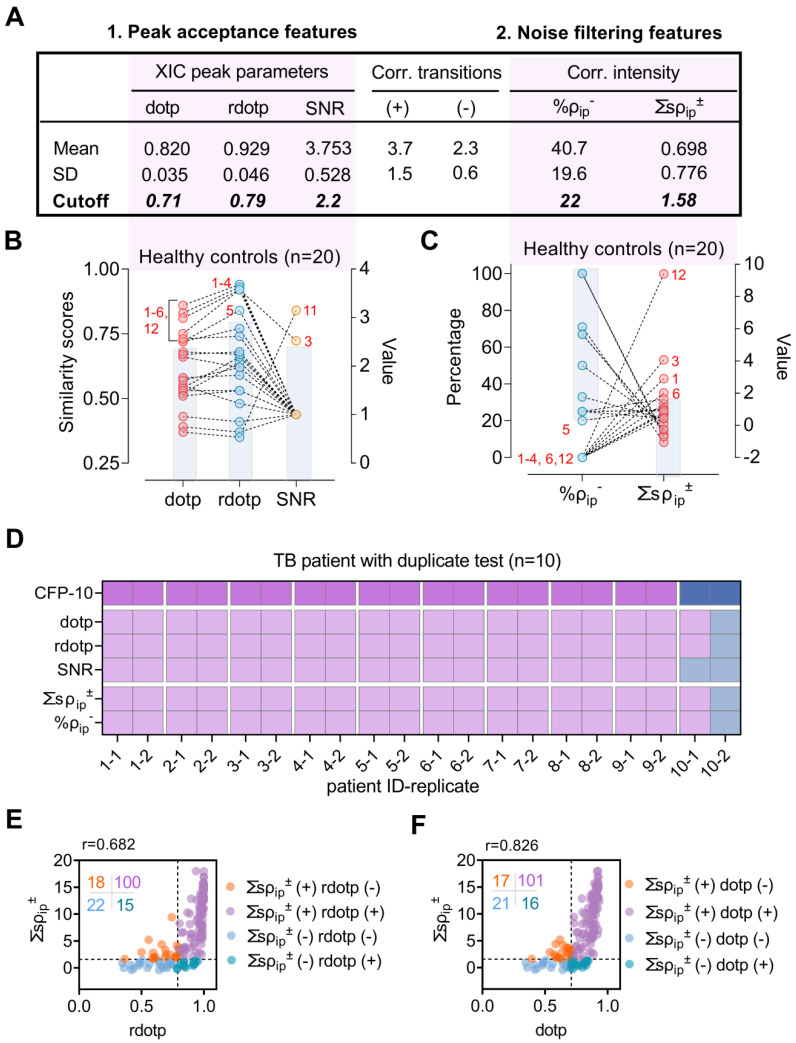
** Performance of peak feature criteria with clinical samples.** (A) Cutoff threshold for peak acceptance features; dotp, rdotp and SNR thresholds employ Mean-3×SD values from the LOD concentration standard (0.5 pM), while those for noise filtering features; the negatively correlated ion pair percentage (%ρ_ip_^-^) and the sum of the significantly correlated ion pair coefficients (∑_s_ρ_ip_^±^) utilize the lower and upper limits of the 95% confidence interval detected in PBS-spiked healthy serum. (B-C) Distribution of values for (B) three peak acceptance and (C) two noise filtering criteria in plasma from 20 healthy donors. Red numbers indicate samples that exceed the exclusion range (blue shaded box) for each feature. (D) CFP-10 positivity, peak feature positivity, and diagnostic information for 10 TB patients with replicate tests. PTB: pulmonary; EPTB, extrapulmonary TB; HIV-TB, HIV and TB co-infection. Blue and purple colors represent for positive and negative results respectively. (E-F) Scatter plot of (E) rdotp and (F) dotp versus the sum of the significant MRM ion pair correlation coefficients (∑ρ_ip_^±^), where the horizontal and vertical dashed lines indicate the positive cut-off thresholds for each parameter, color density indicates overlapping values, and the inset indicates the number of values in each quadrant.

None of these features alone robustly rejected false-positive peaks detected in plasma obtained from 20 healthy donors (**Figure 2B, Table S2**), exhibiting broad individual specificity (median 75%; range 60% to 90%), but together excluded non-specific signal detected in all but one sample (95% specificity). SNR, dotp, and rdotp respectively demonstrated false-positive classification rates of 10, 30, and 35% (**Figure 2B**) in this sample, and one sample was still falsely categorized as CFP-10pep-positive when these samples were screened with all three criteria. Inspection of the extracted ion chromatograms of the four samples with the highest dotp and rdotp values (#1-4, **Figure 2B-C**) revealed co-localization of background noise in the CFP-10pep target, with only one sample (#3) having ion peaks that were aligned and centered in the CFP-10pep elution window (**Figure S6**).

Reproducibility of CFP-10pep peak detection and classification was next evaluated using plasma from 10 TB patients, in a process where each sample was independently processed and analyzed by two individuals. CFP-10pep peak criteria detected positive signal in 9 of these 10 duplicate samples (95% sensitivity). The remaining sample had a low SNR and variable ∑_s_ρ_ip_^±^ and %ρ_ip_^-^ values suggestive of weak or artifactual CFP-10pep signal (**Figure 2D**), likely due to variable capture efficiency during the independent processing of this sample. Mean coefficients of variation were low for dotp and rdotp (3.7% and 3.2%), moderate for ∑_s_ρ_ip_^±^ and %ρ_ip_^-^ (25.4% and 14.9%), and very high for SNR (96.6%), with many replicate SNR values varying by 10- to 100-fold (**Dataset S3**).

### Performance of CFP-10pep criteria in an adult TB cohort

The relationship of these parameters was next evaluated in 155 plasma samples obtained from an adult case-control cohort that contained 12 healthy individuals and 125 TB patients (**Table 1**) enrolled in a multi-site study (see **Methods** for site list). Most TB patients had serum available only after anti-TB treatment initiation (93.6%; 117 of 125), with treatment lengths at plasma collection ranging from 1 to 329 days. Comparison of ∑_s_ρ_ip_^±^ and rdotp values in these samples detected a moderate positive non-parametric correlation (Spearman r = 0.69; **Figure 2E**), with a 65.2% double positive rate. However, ∑_s_ρ_ip_^±^ and rdotp respectively excluded 13.0% (15 of 115) and 15.3% (18 of 118) of the samples judged positive by the other parameter. Similar results were obtained with ∑_s_ρ_ip_^±^ and dotp (**Figure 2F**), but dotp and rdotp exhibited close correlation (Spearman r = 0.95), while SNR revealed variable correlation with the other four parameters (**Figure S7**).

**Figure 3:**
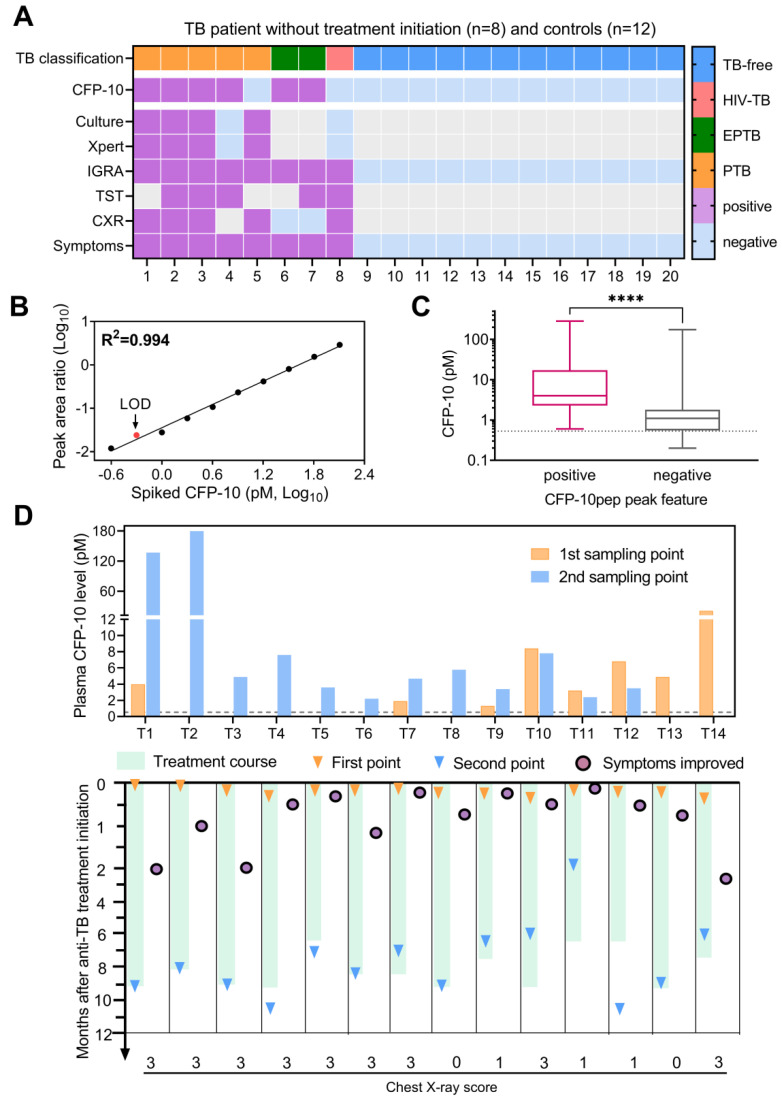
**CFP-10 quantification in adult TB patients. (**A) Plasma CFP-10 positivity and diagnostic information for adult TB patients without anti-TB treatment. Gray indicates missing data. (B) CFP-10 calibration curve. (C) Plasma CFP-10 level in the CFP-10pep peak feature-positive (n = 86) and -negative (n = 69) groups. The Mann-Whitney test difference significance of CFP-10 quantity in the two groups is ****p < 0.0001. Box plots indicate the median and interquartile range and error bars indicate the minimum and maximum values. (D) Quantification of CFP-10 changes in adult TB patients after anti-TB treatment initiation. Dashed lines in C-D indicate the assay LOD. Patients T1-T6 and T10-14 were diagnosed by microbiologic results and T7-T9 were diagnosed by clinical or histologic findings, and assigned chest x-ray severity scores (0-3: normal, mild, intermediate, severe phenotype), as detailed in **Dataset S4**.

CFP-10pep peak criteria detected positive signal in most (75%; 6 of 8) plasma samples collected from TB patients before anti-TB treatment but in no (0 of 12) samples obtained from the healthy controls (**Figure 3A**). CFP-10pep/IS signal detected in these samples was then compared to a standard curve generated by spiking healthy commercial donor plasma serial dilutions of recombinant CFP-10 and a constant amount of IS peptide. This calibration curve revealed good linearity (Pearson R^2^=0.995, **Figure 3B**) over a broad CFP-10 concentration range (0.25 - 128 pM), with an estimated 0.53 pM and limit of detection (LOD) (**Table S3**). All samples judged CFP-10pep positive by CFP-10pep acceptance criteria had CFP-10pep/IS values equal to or greater than this LOD (**Figure 3C** and **Dataset S4**). However, the CFP-10pep/IS ratio alone was not a good predictor of CFP-10pep signal since most samples judged CFP-10pep negative by the CFP-10pep peak criteria (83%; 57 of 69) exceeded the CFP-10/IS LOD, implying that there was significant noise from the off-target ions in the LC-MS target window.

Most TB cases with serial plasma available (77.8%; 14 of 18) were CFP-10pep criteria-positive pre- and/or post-treatment initiation, and all criteria-positive samples had CFP-10/IS ratios above the established LOD (**Figure 3D** and** Dataset S5**). Surprisingly, most (75%; 6 of 8) TB patients with CXR scores indicating high-grade lung infiltration and microbial evidence of TB had low or undetectable baseline CFP-10 levels that increased after treatment initiation. Conversely, most (83%; 5 of 6) TB patients with CFP-10 levels >2 pM at baseline revealed stable or decreased CFP-10 levels after treatment initiation. Most CFP-10 increases occurred in men (7 of 9), and most stable or decreasing CFP-10 values occurred in women (4 of 5), suggesting a possible gender effect 31, but this observation needs to be replicated in an independent study powered to detect such effects. CXR scores have been used to predict anti-TB treatment response, as indicated by 2-month smear status in adults with smear-positive PTB^32^. Consistent with the results of this study, our data suggested that a high-grade CXR was correlated with an increase of CFP-10 after treatment.

### Performance of the CFP-10pep criteria in two pediatric TB cohorts

**Figure 4:**
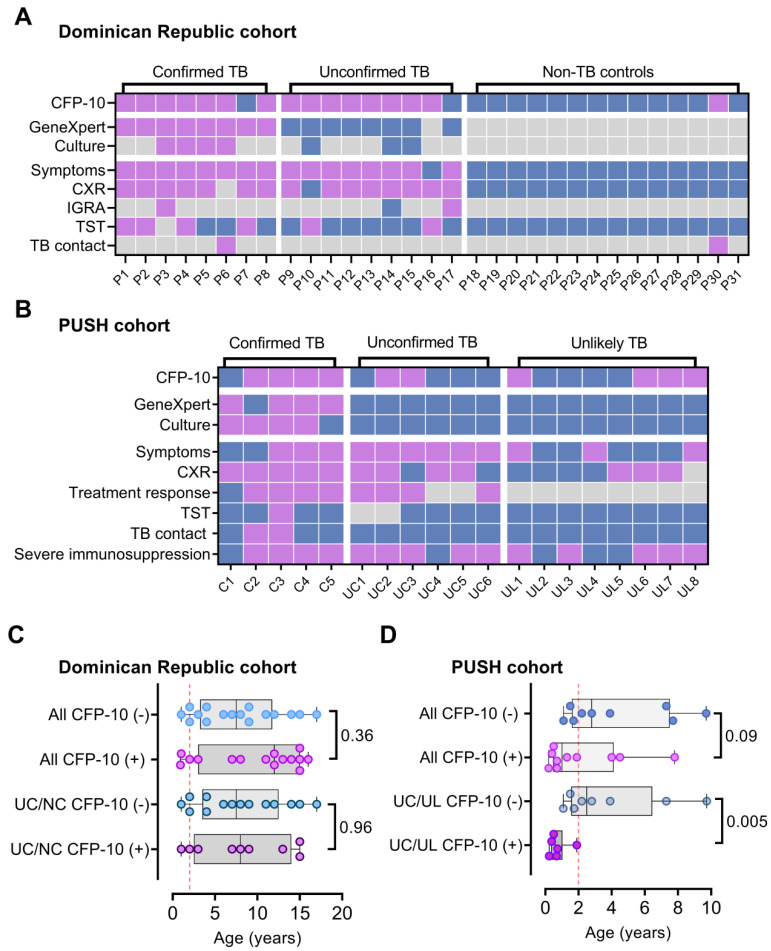
** CFP-10pep diagnosis of TB in pediatric patients with or without HIV infections.** (A-B) Plasma CFP-10 positivity and diagnostic information for children in the (A) Dominican Republic cohort and the (B) Kenyan HIV-infected PUSH cohort. Blue, purple and grey squares indicate negative, positive, and missing data respectively. CXR, chest X-ray test. TST, tuberculin skin test. (C-D) Age distribution of CFP-10 children with positive and negative results in the (C) Dominican Republic cohort and (D) Kenyan PUSH cohort, indicating Mann-Whitney U test p-values for each comparison, where p < 0.05 is considered significant. UC/UL: unconfirmed TB /unlikely TB children; UC/NC: unconfirmed/non-TB contact children. Dashed line in (C-D) indicate a threshold at 2 years-of-age.

Disease diagnosis, including TB diagnosis, can be more challenging in children than adults, and in individuals living with HIV. We therefore evaluated the ability of serum CFP-10pep signal to diagnose pediatric TB cases in small, predominantly HIV-uninfected (96.8%; 30 of 31 cases) pediatric cohort enrolled in the Dominican Republic. This analysis detected CFP-10pep signal in pre-treatment serum from most microbiologically confirmed TB (87.5%; 7 of 8) and clinically diagnosed TB cases (75%; 7 of 9; **Figure 4A**). CFP-10pep signal was not detected in serum from most children in the non-TB group (92.9%; 32 of 33), all of whom lacked evidence of *Mtb* infection by negative tuberculin skin test results (**Dataset S6**). The only child in the non-TB cohort who had a positive result was a household contact of an TB patient, implying this child could have had incipient TB. However, follow-up data was not available to address this question.

Individuals with immature or compromised immune systems can also be difficult to diagnose by standard microbiologic approaches, since deficient immune responses can permit *Mtb* bacilli to migrate beyond the lungs and thus reduce their abundance in respiratory samples used in conventional diagnostic approaches. Serum from a cohort of hospitalized HIV-infected children aged <12 years with suspected TB (PUSH cohort, see **Methods**) was therefore analyzed to evaluate CFP-10pep diagnostic performance in a more challenging TB patient cohort. These children were segregated into three categories: confirmed TB (microbiologic evidence of TB); unconfirmed TB (no microbiologic evidence but at least two other criteria: TB-associated symptoms, TB-consistent CXR, close TB exposure or immunologic evidence of *Mtb* infection, or positive TB treatment response); or unlikely TB (negative for confirmed and unconfirmed TB criteria).

CFP-10pep signal was detected in pre-treatment serum from 50% to 83% of the children in these three groups (**Figure 4B** and **Dataset S7**). Most unconfirmed TB cases had CFP-10pep positive samples (83.3%; 5 of 6), and the single CFP-10pep negative confirmed TB case died shortly after anti-TB treatment initiation, precluding further evaluation. Surprisingly, however, CFP-10pep positive samples were not enriched in the unconfirmed TB versus unlikely TB groups (33.3% versus 50%), as might be predicted due to the greater evidence for TB in the former group, and the reason for conversely low and high detection rates in these groups is not clear. All children with CFP-10pep positive serum met the criteria for severe immunosuppression and demonstrated symptom improvement when administered anti-TB treatment, although children in both categories were also classified as CFP-10pep negative. However, while children in the DR cohort who had CFP-10pep positive and negative samples did not differ by age, unconfirmed and unlikely PUSH cohort TB cases who had CFP-10pep positive samples were markedly younger than their CFP-10pep negative counterparts (**Figure 4C**-**D**). This strong age bias is consistent with the observation that age is the most important factor in disease progression after primary *Mtb* infection in children with functional or compromised immune systems and that very young children (aged <2 years) co-infected with HIV and *Mtb* have high risk of TB progression 33, 34.

### Differentiation of TB infection and active TB by CFP-10pep assay

**Figure 5:**
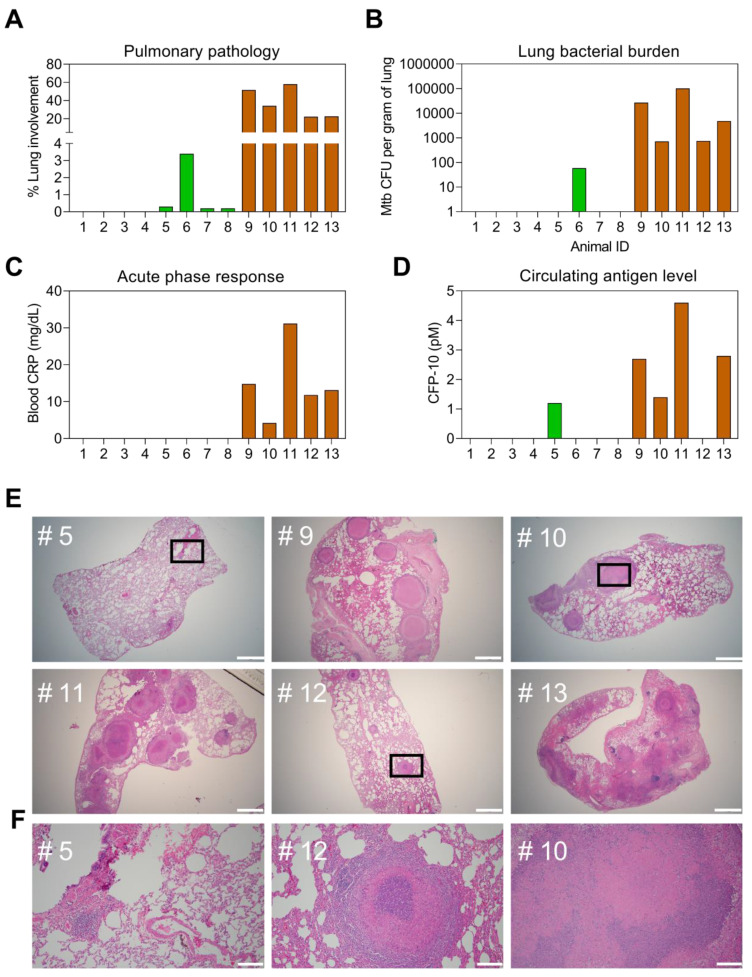
** Correspondence of CFP-10 level with pathology in a rhesus macaque TB model.** Pulmonary pathology at necropsy was evaluated as the (A) percentage of lung involvement and (B) lung *Mtb* burden expressed in colony-forming units (CFUs). Plasma levels of (C) C-reactive protein (CRP) and (D) CFP-10 at necropsy were evaluated as markers of active infection and TB disease. NHPs evaluated were not exposed to *Mtb* (IDs 1-4, white bars, or exposed to low or high aerosol doses (200 vs 10 CFUs, see **Methods** for details) and respectively developed latent TB infections (TBI, IDs 5-8, green bars) or active TB disease (TB, IDs 9-13, orange bars), as confirmed by subsequent evaluations. (E) H&E staining of lung sections from NHP animals with latent TB infections or TB disease, where selected areas (black squares) were reviewed at higher magnification to evaluate regions with potential granulomas. White lines indicate (E) 1, 000 µm and (F) 100 µm scale bars.

Evidence suggests that CFP-10 secretion may be regulated by at distinct stages of infection, and thus may serve as a means to distinguish TB cases from asymptomatic TB infections (TBI) that do not cause disease 35, but which are estimated to affect ~25% of the global population 36. Since it is difficult to distinguish TBI from subclinical TB in patients 5, 35, 37, plasma samples obtained from a non-human primate (NHP) model designed to mimic TBI and active TB (ATB) 19 were evaluated to detect potential differences in plasma CFP-10pep expression among these groups. NHPs analyzed in this study were exposed to aerosols containing low or high doses of *Mtb* to induce TBI or ATB, or not exposed to* Mtb* aerosols to provide an *Mtb*-naïve control group (**Dataset S8**), respectively. NHPs in the TBI group revealed immunologic evidence of *Mtb* infection (positive TST scores) a month after *Mtb* exposure and maintained asymptomatic TBI-like infections throughout the study duration (~22 weeks), whereas NHPs in the ATB group developed active pulmonary disease characterized by weight loss, fever, and elevated serum C-reactive protein (CRP) and chest radiograph scores. NHPs in the TBI group also revealed markedly reduced evidence of lung pathology, *Mtb* bacillary burden, and systemic inflammation as determined by plasma levels of CRP than NHPs in the ATB group (**Figure 5A-C**). All these results corresponded with serum CFP-10 levels measured in the plasma samples of these groups (**Figure 5D** and** Dataset S9**). Plasma CFP-10 levels also tended to correspond with granuloma size, abundance, and structure in lung tissue sections. NHPs in the ATB groups with higher plasma CFP-10 levels tended to have more and larger granulomas (**Figure 5D-E**: 9, 11, and 13) than those with lower or undetectable plasma CFP-10 levels (**Figure 5D-F:** 10 and 12). Further, NHP 12, which had an undetectable serum CPF-10 level, exhibited a distinctive granuloma histology pattern characterized by a densely staining center mass that resembled that observed in the lone TBI case with detectable CFP-10 signal (**Figure 5F**: 5).

## Discussion

Our IP-MRM-MS workflow is intended to identify peptides from pathogen-derived proteins that circulate at low concentration and may be subject to masking effects. Such biomarkers may not be detectable by either immunoassay or MRM-MS alone due to confounding factors and instrument limitations. For example, host factors may interact with such biomarkers to mask their recognition by standard immunoassays. Some immunoassays (e.g., HIV-1 p24 and thyroglobulin assays) include steps designed to disrupt such complexes to allow assay antibodies to compete for biomarker binding, but may have limited sensitivity at low concentrations 38-41. Immuno-MRM assays for thyroglobulin, which normally circulates at relatively high level (20-25 ng/mL), are thus being developed by two large clinical testing laboratories 42. Similarly, MRM-MS can be used to improve the specificity of target detection, but its clinical utility is limited by the composition of the analysis sample and relative biomarker abundance. Standard serum and plasma samples contain multiple factors that can degrade the performance of an LC column, and need to be removed by pre-clearance procedures that do not also deplete the target biomarker. Biomarker targets must also compete with a diverse array protein for limited LC column binding capacity in these assays, and thus low concentration biomarkers may be undetectable without highly selective affinity enrichment or pre-fractionation procedures.

Immunoaffinity MRM MS (IA-MRM-MS) assays that detect pathogen-derived peptides as biomarkers of infectious disease require additional refinements beyond those employed for human serum proteins, due to their more stringent sensitivity and specificity requirements. Such assay may need to differentiate among similar peptides produced by related pathogens to allow species-specific identification, and thus peptide selection is constrained by potential sequence homology among pathogens capable of producing human infections that present with similar clinical findings. Further, pathogen-derived biomarkers may be present at very low concentrations, particularly during the early stage of an infection, so that disease diagnosis may depend upon accurate determination of whether weak MS signal represents the target peptide, non-specific signal, or background noise.

The design of IA-MRM-MS assays that target human proteins tends to be less complicated since these assays tend to evaluate changes in relatively level of more abundant host protein-derived target peptides rather than differentiating low level *de novo* expression from absence of expression. Biomarker peptides for these assays are selected from the human proteome using five criteria: 1) preferred length (8-14 amino acids) for antibody generation efficiency; 2) preferred mass (>800 Da), for efficient MS detection; 3) minimum antigenicity (Hopp-Woods hydrophilicity score of -0.5 to +0.5); 4) chemical stability (no Met, Trp, or Cys to reduce potential effects); and 5) uniform composition (no post-translational modifications or allelic variations 43. However, virulence factors secreted by bacterial pathogens can be small, present at low abundance, and conserved among related species, so our approach uses a less restricted size range to allow greater coverage of the limited peptidome of these protein targets. It also includes criteria for MS detection efficiency and species-specificity/selectivity not required for host-derived targets, which are typically present at high pg/mL to low ng/mL concentrations 42, 44 and thus less subject to interference than secreted virulence factors that may be present at or below pg/mL concentrations.

Screening approaches are also required to distinguish low abundance peptide biomarker signal - typically presented as peak height or area - from peptide contaminants and MS noise that can produce false-positive signals. SNR and sample/IS peak area ratios from negative control sample(s) are often used to set minimum thresholds for positive signal 40, 45, 46, but are not sufficient to reliably distinguish target peaks from noise at low target concentrations. Interference peaks with high SNRs can arise from contaminant peptides that have mass-to-charge ratios (m/z) similar to target peptides, but different amino acid sequences 43, or from electronic noise detected during sample analysis. Ratios determined among at least three product ions are also used to detect and quantify target peaks, since product ion intensity ratios are independent of analyte concentration over the linear operating range of a MS system. However, these ions are susceptible to misidentification leading to inaccurate identification and quantification.

Peak feature criteria selected in this study demonstrated better specificity than traditional peak intensity cutoff settings in multiple independent populations, including diagnostically challenging cohorts with HIV-TB co-infection. These criteria demonstrated good specificity in distinguishing true peptide biomarker signal from coeluting noise, which was not possible using peak identification criteria by the mProphet semi-supervised learning algorithm to control false discovery rate using multiple decoy transitions and demonstrated good reproducibility for target peak identification in replicate samples. This method thus appears to provide a robust means for peak classification and biomarker quantification that should be readily transferable to other biomarker targets 47.

## Supplementary Material

Supplementary methods, figures, tables, and dataset legends.

Supplementary dataset 1.

Supplementary dataset 2.

Supplementary dataset 3.

Supplementary dataset 4.

Supplementary dataset 5.

Supplementary dataset 6.

Supplementary dataset 7.

Supplementary dataset 8.

Supplementary dataset 9.

## Figures and Tables

**Table 1:** Demographics and clinical characteristics of the adult TB study.

	Total	Controls	TB	HIV-TB
Participants, n	137	12	111	14
Age, yrs	35 (26, 44)	44 (30, 52)	34 (26, 42)	31 (27, 45)
Female sex	56 (40.9)	10 (83.3)	41 (36.9)	5 (35.7)
BCG vaccinated	99 (72.3)	2 (16.7)	86 (77.5)	11 (78.7)
Confirmed TB localization				
PTB	114 (83.2)	--	105 (94.6)	9 (64.3)
EPTB	7 (5.1)	--	4 (3.6)	3 (21.4)
PTB and EPTB	4 (2.9)	--	2 (1.8)	2 (14.3)

Data represent median (interquartile range) or n (%). BCG: Bacille Calmette-Guérin; PTB: pulmonary TB; EPTB: extrapulmonary TB.
