# Supplementary material for: Assay design for unambiguous identification and quantification of circulating pathogen-derived peptide biomarkers
Source: Theranostics. 2022 Mar 21;12(6):2948–62. doi: 10.7150/thno.70373 (PMC8965485; doi:10.7150/thno.70373)
Supplement: Supplementary file 1 — Supplementary methods, figures, tables, and dataset legends. [file thnov12p2948s1.pdf]

Supplemental Information for

Assay design for unambiguous identification and quantification of circulating pathogen-derived peptide biomarkers

Qingbo Shu[1]†, Shan Liu[2, 1]†, Tonino Alonzi[3], Sylvia M. LaCourse[4], Dhiraj Kumar Singh[5], Duran Bao[1], Dalton Wamalwa[6], Li Jiang[2,7], Christopher J. Lyon[1], Grace John-Stewart[4], Deepak Kaushal[5], Delia Goletti[3], Tony Hu[1]*

1 Center for Cellular and Molecular Diagnostics, Department of Biochemistry and Molecular Biology, School of Medicine, Tulane University, New Orleans, Louisiana, USA.

2 Sichuan Provincial Key Laboratory for Human Disease Gene Study, Department of Medical Genetics, Department of Laboratory medicine, Sichuan Academy of Medical Sciences & Sichuan Provincial People's Hospital, University of Electronic Science and Technology, Chengdu, China.

3 Translational Research Unit, National Institute for Infectious Diseases Lazzaro Spallanzani-IRCCS, Rome, Italy.

4 Departments of Medicine, Division of Allergy and Infectious Diseases, and Global Health, University of Washington, Seattle, USA.

5 Southwest National Primate Research Center, Texas Biomedical Research Institute, San Antonio, Texas, USA.

6 Department of Pediatrics and Child Health, University of Nairobi, Nairobi, Kenya.

7 Department of Laboratory Medicine, Sichuan Academy of Medical Sciences and Sichuan Provincial People's Hospital, Chengdu, China.

*corresponding author: Tony Hu. tonyhu@tulane.edu

Table of Content

Item	Name	Page #
Supplemental Methods	Diagnosis of adult and pediatric TB patients	3-4
Supplemental Methods	StageTip clean-up of peptide eluent	4
Supplemental Methods	Standard curve for CFP-10 quantification	5
Supplemental References	Supplemental References	5-6
Figure S1	Determination of the retention time window for MRM time series extraction	7-8
Figure S2	Target peptide selection based on sequence specificity and immunogenicity	9-10
Figure S3	Selection of CFP-10pep MRM-MS transition ions	11-12
Figure S4	Peak recognition and differentiation of noise from signal by transition correlation analysis	13-14
Figure S5	Peak recognition and differentiation of noise from signal by transition correlation analysis	15-16
Figure S6	CFP-10pep assay results from four healthy donors with dotp and rdotp values above the selected cutoffs for these parameters (#1-4 in Fig. 2a-b)	17
Figure S7	Spearman correlation analysis of peak features	18
Table S1	CFP-10pep peak features in triplicates of serum spiked with 0.5 pM CFP-10 or PBS	19
Table S2	Summary of CFP-10pep criteria detected in 20 healthy control samples	20
Table S3	Estimate of the limit of detection of the CFP-10pep MRM assay	21
Dataset S1	CFP-10pep mouse monoclonal antibody affinity and immunoprecipitation yield	Separate file
Dataset S2	Predicted interference ions for each transition of CFP-10pep	Separate file
Dataset S3	Repeating test results of CFP-10pep assay in 10 plasma samples	Separate file
Dataset S4	CFP-10pep assay result in 155 clinical plasma samples	Separate file
Dataset S5	CFP-10pep assay and clinical diagnosis tests result for 18 adult TB patients during anti-TB treatment	Separate file
Dataset S6	Demographics and clinical information of 31 participants in Dominican Republic cohort	Separate file
Dataset S7	Clinical information and diagnosis of 19 patients from PUSH pediatric cohort	Separate file
Dataset S8	Demographics and characterization of rhesus macaques infected with *Mtb*	Separate file
Dataset S9	CFP-10pep assay result in the plasma samples from NHP model of *Mtb* infection	Separate file

Diagnosis of adult and pediatric TB patients.

Microbiologically confirmed TB cases were identified by a positive result for at least one of three tests: *Mtb* culture (sputum, bronchoalveolar lavage, pleural fluid, or an abscess specimen); *Mtb*-specific RNA amplification (TRCReady MTB, Tosoh, Japan) and/or *Mtb*-specific DNA amplification (laboratory PCR for the *Mtb* IS6110 gene; GeneXpert (Cepheid) assay; or GenoType MTBDRplus assay (Hain Lifescience)); and/or histopathological findings and presence of acid-fast bacilli in tissue specimens. Clinically identified TB cases were diagnosed based on clinical and radiologic criteria, including appropriate response to TB-specific therapy. Extrapulmonary TB (EPTB) was defined as TB disease in any body region other than the lungs (https://www.cdc.gov/tb/topic/basics/glossary.htm). Diagnosis was performed by *Mtb* culture of respiratory specimens or GeneXpert or GenoType MTBDRplus analysis of sputum, pleural fluid, lymph nodes aspirate, or other specimens, or histology. In patients with negative microbiologic results, diagnosis of EPTB was determined by clinical means. EPTB patients had symptoms (fever, loss of appetite, weight loss, local pain) and signs (positive TST or IGRA; radiological images) that remitted after completion of specific anti-TB therapy. Identified TB cases were treated with first line drugs, as indicated by the updated international guidelines (1), and followed for 2 months after treatment completion. TB treatment outcomes were evaluated by (1) sputum culture conversion (for pulmonary TB cases), (2) improvement in clinical findings, and (3) improvement in radiologic lesions.

Healthy controls were individuals with low risk for *Mtb*-exposure, who lacked clinical and radiological signs consistent with active TB disease, where potential TBI was excluded based on a negative IGRA result.

Children enrolled in this study were malnourished, severely immunosuppressed, and had 22% overall mortality. All children were systematically screened for TB symptoms and TB exposure. Positive tuberculin skin tests were defined by indurations >5mm. At enrollment, all children provided sputum or gastric aspirates for GeneXpert MTB/RIF analysis and culture, urine for lipoarabinomannan (LAM) antigen testing, and stool for GeneXpert MTB/RIF testing, irrespective of TB symptoms, with a second sputum/gastric aspirate obtained for a second *Mtb* culture (2). Children had serum and plasma collected and cryopreserved at enrollment, and at 1, 2, 4, 12, and 24 weeks post-enrollment. Chest radiographs were read by a radiologist to identify findings suggestive of TB using standardized reporting forms developed by the South African Tuberculosis Vaccine Initiative (3). Diagnostic results were available to study clinicians, who initiated TB treatment at their discretion per Kenyan guidelines. Children were categorized as Confirmed, Unconfirmed, or Unlikely TB based on international consensus clinical case definitions (4).

StageTip clean-up of peptide eluent

StageTips (5) were made by packing 200 µL pipette tips with four layers of Empore C8 solid phase extraction disk (Cat. No. 2214-C8, 3M), washed by 25°C centrifugation at 1000g for 3 min with 50 µL of 0.1% (v/v) TFA acetonitrile solution and 50 µL of 0.1% (v/v) TFA. Each peptide sample was split and loaded onto two StageTips, and captured peptides were eluted with 50 µL of 0.1% (v/v) TFA acetonitrile solution. Paired eluents were combined, dried by vacuum concentration, re-dissolved with 8 µL of sampling buffer containing 0.1% (v/v) formic acid, 2% (v/v) acetonitrile, and centrifuged at 25°C and 21,000 g, for 10 min. After centrifuging the

reconstituted samples at 20 °C and 21,000g for 15 minutes, the supernatant is transfer into an MS sample vial.

Standard curve for CFP-10 quantification

A standard curve used to quantify CFP-10 concentrations in serum was built by performing two-fold serial dilutions of recombinant CFP-10 in healthy human serum over a range of 0.25-128 pM. These samples were then trypsin digested, spiked with a constant amount of synthetic CFP-10pep stable-isotope-labeled internal standard peptide, subjected to immunoaffinity enrichment, and then analyzed by MRM MS. Linear curves and correlation coefficients for the relationship between the CFP-10pep/IS peak area ratio and CFP-10 concentration were generated using GraphPad Prism (version 8.4.3, GraphPad Software). Since peaks detected in the 0.25 pM standard did not meet CFP-10 peak detection criteria (**Table S3**), the peak area ratio of this this false-negative sample was analyzed 10 times and mean of this value plus three times its standard deviation was used to calculate the CFP-10pep limit of detection (0.53 pM) when evaluated by the standard curve equation.

Supplemental References

1. Nahid P, Dorman SE, Alipanah N, Barry PM, Brozek JL, Cattamanchi A, et al. Official American Thoracic Society/Centers for Disease Control and Prevention/Infectious Diseases Society of America Clinical Practice Guidelines: Treatment of Drug-Susceptible Tuberculosis. Clin Infect Dis. 2016;63(7):e147-e95.
2. LaCourse SM, Pavlinac PB, Cranmer LM, Njuguna IN, Mugo C, Gatimu J, et al. Stool Xpert MTB/RIF and urine lipoarabinomannan for the diagnosis of tuberculosis in hospitalized HIV-infected children. AIDS. 2018;32(1):69-78.
3. Graham SM, Ahmed T, Amanullah F, Browning R, Cardenas V, Casenghi M, et al. Evaluation of tuberculosis diagnostics in children: 1. Proposed clinical case definitions for classification of intrathoracic tuberculosis disease. Consensus from an expert panel. J Infect Dis. 2012;205 Suppl 2:S199-208.

4. Graham SM, Cuevas LE, Jean-Philippe P, Browning R, Casenghi M, Detjen AK, et al. Clinical Case Definitions for Classification of Intrathoracic Tuberculosis in Children: An Update. Clin Infect Dis. 2015;61Suppl 3:S179-87.
5. Rappsilber J, Mann M, Ishihama Y. Protocol for micro-purification, enrichment, pre-fractionation and storage of peptides for proteomics using StageTips. Nature Protocols. 2007;2(8):1896-906.

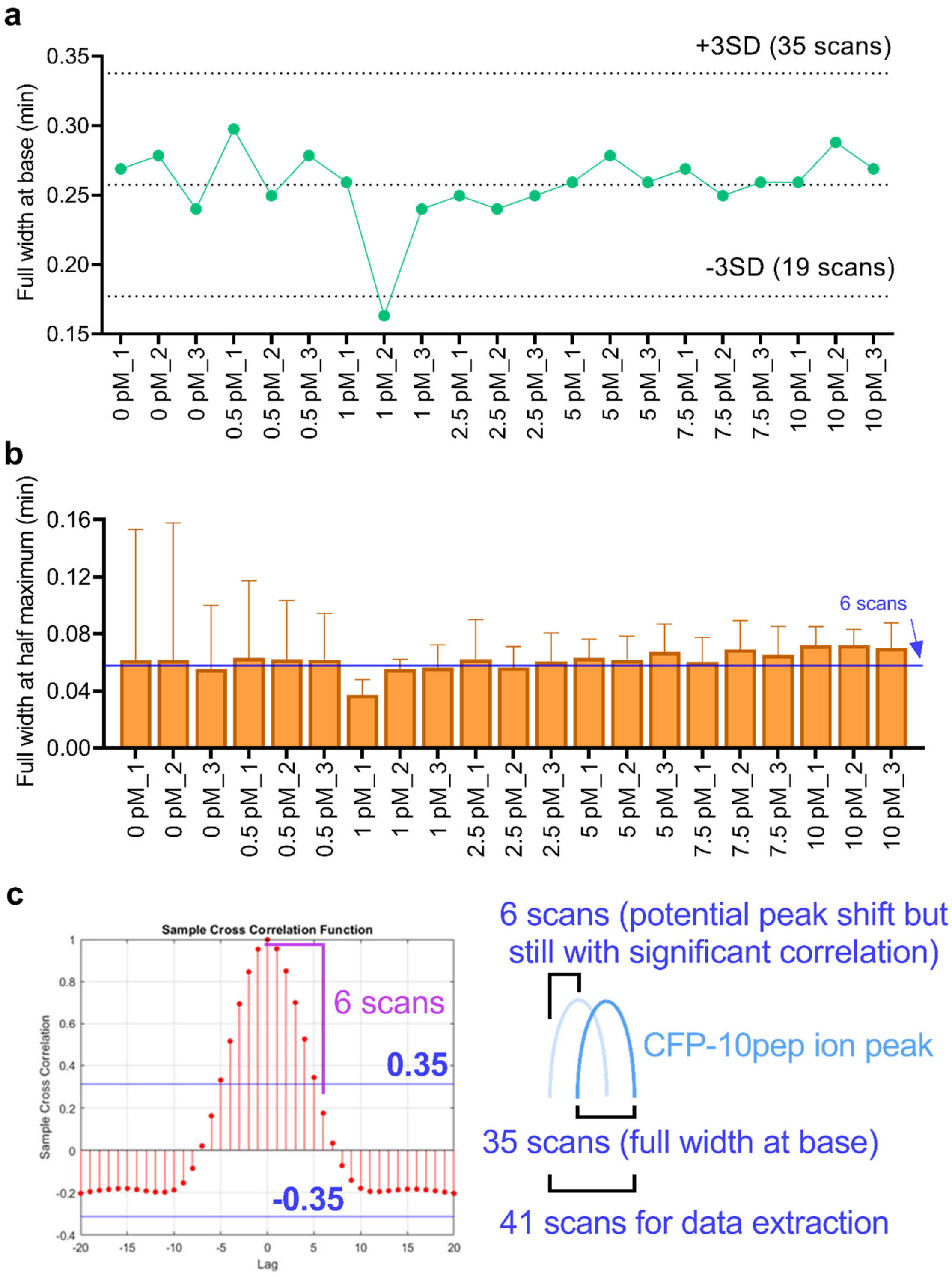

Figure S1. Determination of the retention time window for MRM time series extraction. **a** Full width at base of CFP-10pep precursor ion. **b** The average full width at half maximum of the

seven ions in healthy human serum spiked with 0-10 pM of recombinant CFP-10 protein prior to trypsin digestion, indicating the 3-fold standard deviation (SD) of this measure as dashed lines and individual values for each concentration standard. The scan numbers are calculated based on the fixed cycle time of each scan as 0.0096 min. **c** Evaluation of the elution window as the number of scans for MRM data extraction and analysis. Cross-correlation was used to assess the potential peak shift that can be significantly correlated (absolute value of coefficient>0.35). The CFP-10pep elution window (41 scans) was defined by its maximum full width at base (35 scans) plus the potential shift as 6 scans.

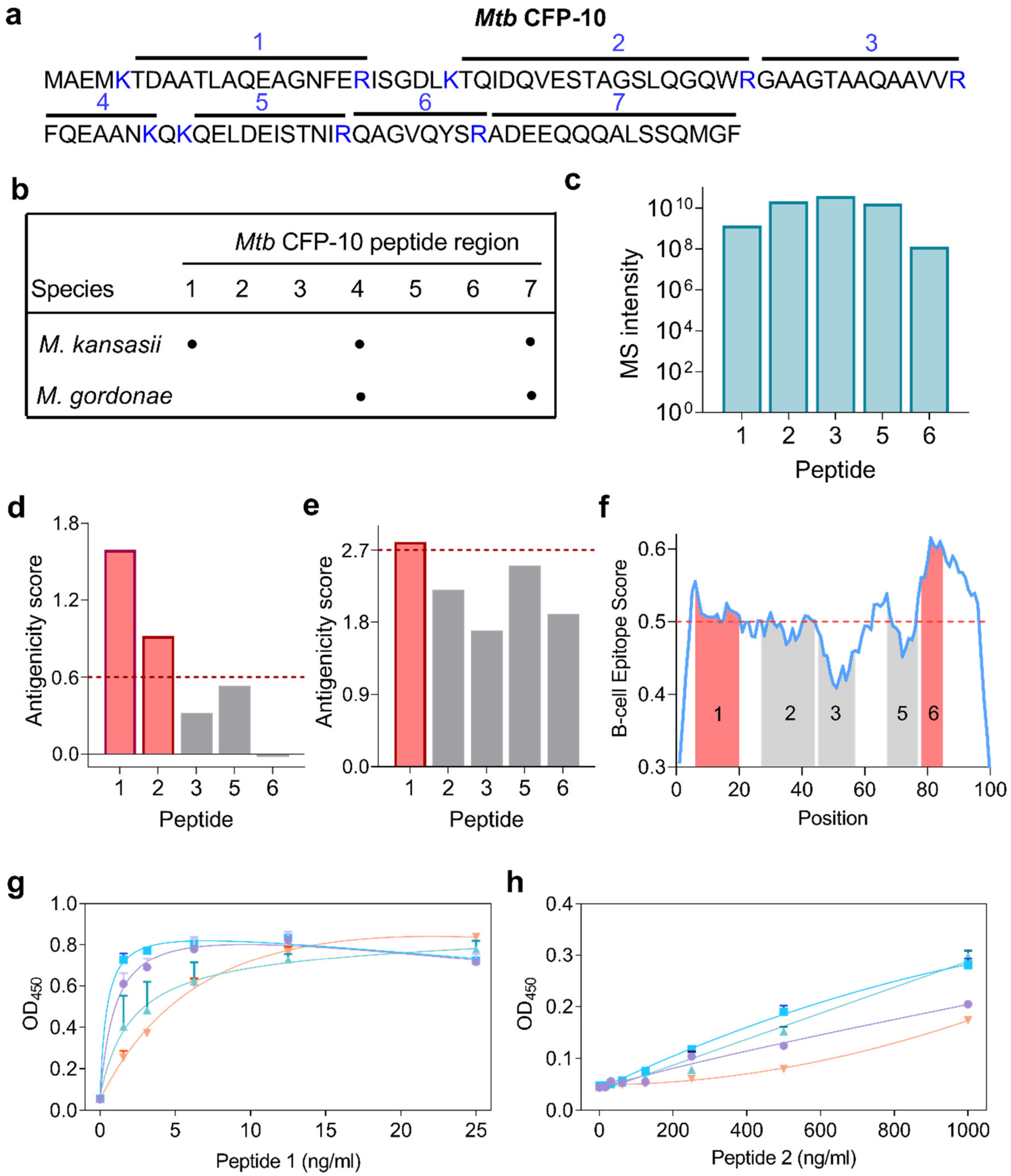

Figure S2. Target peptide selection based on sequence specificity and immunogenicity. a Map of *Mtb* CFP-10 tryptic peptides ≥ 7 amino acids. **b** *Mtb* CFP-10 peptide matches with NTM species responsible for >80% of pulmonary mycobacterial infections. **c** Precursor ion intensities for *Mtb*-specific/selective tryptic peptides derived from a recombinant *Mtb* CFP-10 protein digest. **d-f**

Predicted antigenicity of candidate peptides estimated by algorithms from (**d**) GenScript and (**e**) Thermo Scientific, and (**f**) the B-cell epitope score (blue line) of each amino acid in the full-length CFP-10 sequence. Peptides and their sequence regions that score above or below the cut-off values (dashed gray lines) of the indicated algorithms are shaded red or gray, respectively. **g-h** Relative ELISA signal detected upon incubation of immune serum from four rabbits (colored lines) immunized against (**g**) peptide 1 or (**h**) peptide 2 with serial dilutions of their target peptides.

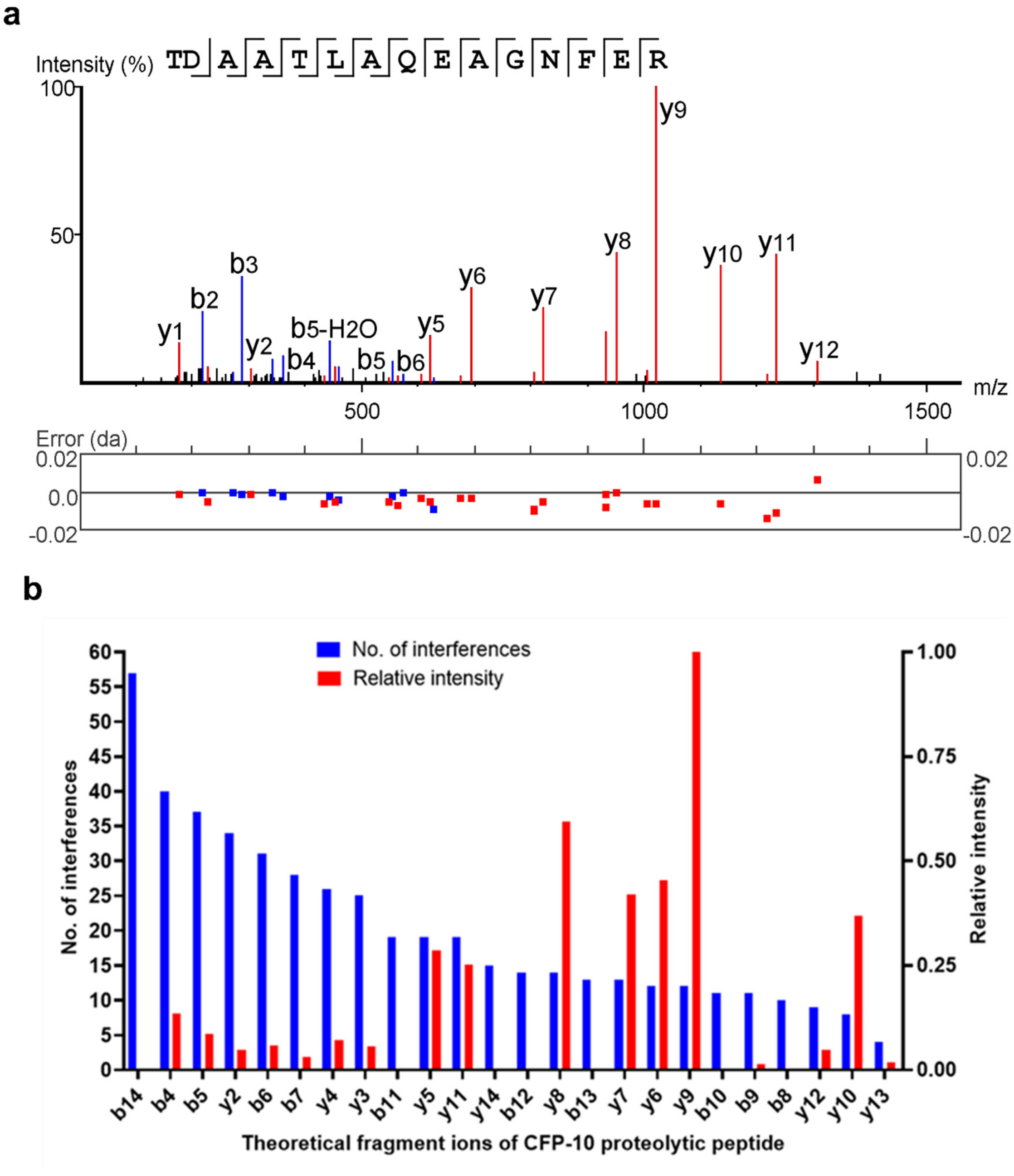

Figure S3. **Selection of CFP-10pep MRM-MS transition ions. a** Annotated tandem MS spectrum data for *Mtb* CFP-10pep, with mass errors shown below the spectrum. **b** Comparison of the peak intensities versus the number of theoretical CFP-10pep fragment ion interferences. SRMCollider software was used to predict potential interferences for each theoretical CFP10pep MRM transition when setting the human proteome as the peptide background. Seven fragment

ions (y5-y11) were selected as MRM Q3 ions based on their relative signal and number of potentially inferring ions.

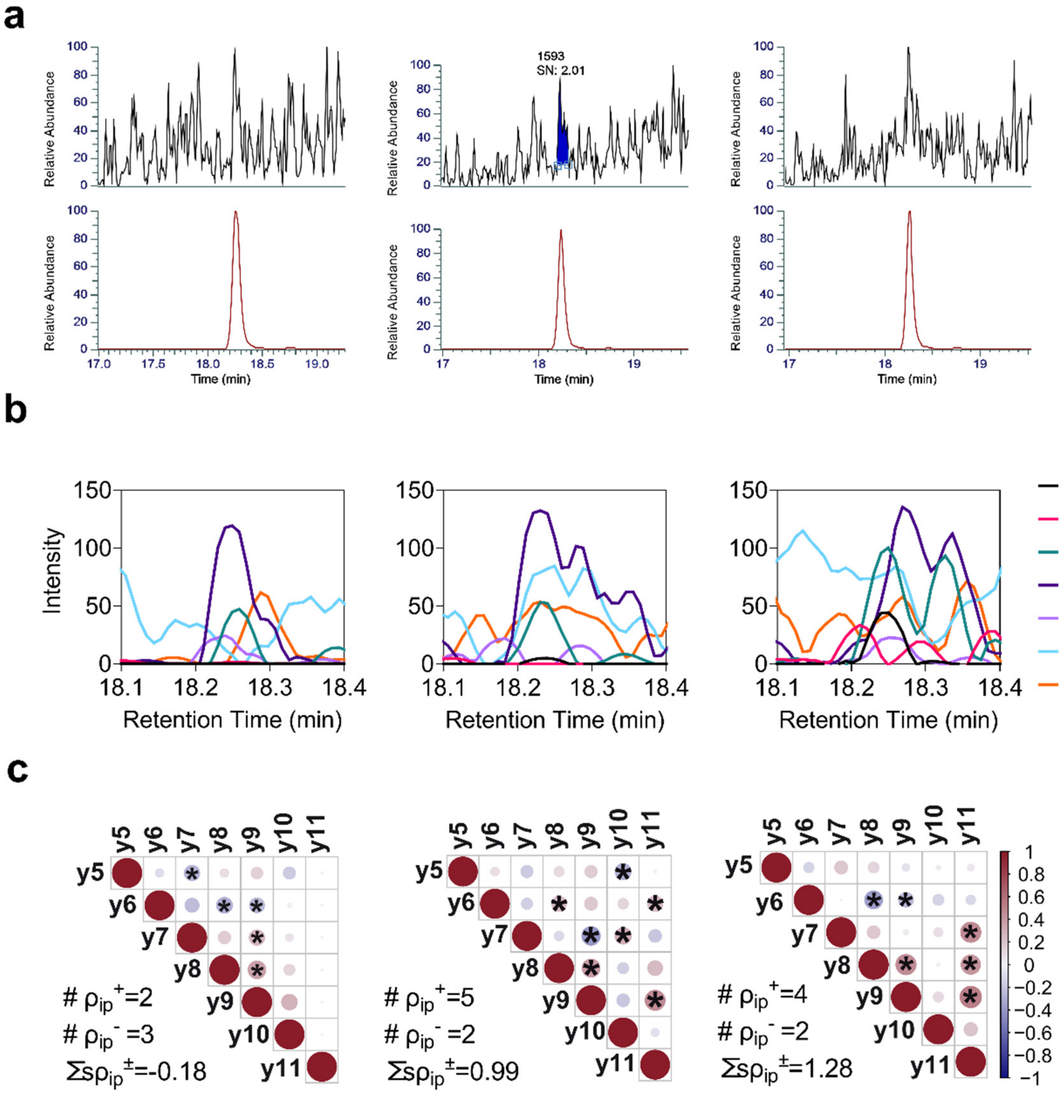

Figure S4. **Peak recognition and differentiation of noise from signal by transition correlation analysis. a** Extracted ion chromatographs for (upper panel) CFP-10pep and (lower panel) its heavy-isotope-labeled IS peptide in affinity enriched samples from three replicates of trypsin-digested healthy human serum spiked with 12.5 nM of the IS peptide. Recognized peaks (blue) are labeled with their signal-to-noise ratio (SN). **b** Targeted retention time window extracted ion chromatographs for the seven selected transition ions in the blank serum samples in (**a**). **c** Kendall rank order correlation coefficient matrix for the seven transitions in blank serum samples.

Correlation coefficients are color coded, and the circle size represented for their significance p value. Asterisks indicate the correlation coefficients that significantly differ from zero ($p<0.05$), as determined by the Kendall tau value. The number of positive and negative correlated transition ions (# ρ_{ip}^{+}, # ρ_{ip}^{-}), and the sum of all significant coefficients ($\sum_{s}\rho_{ip}^{\pm}$) are indicated on each array.

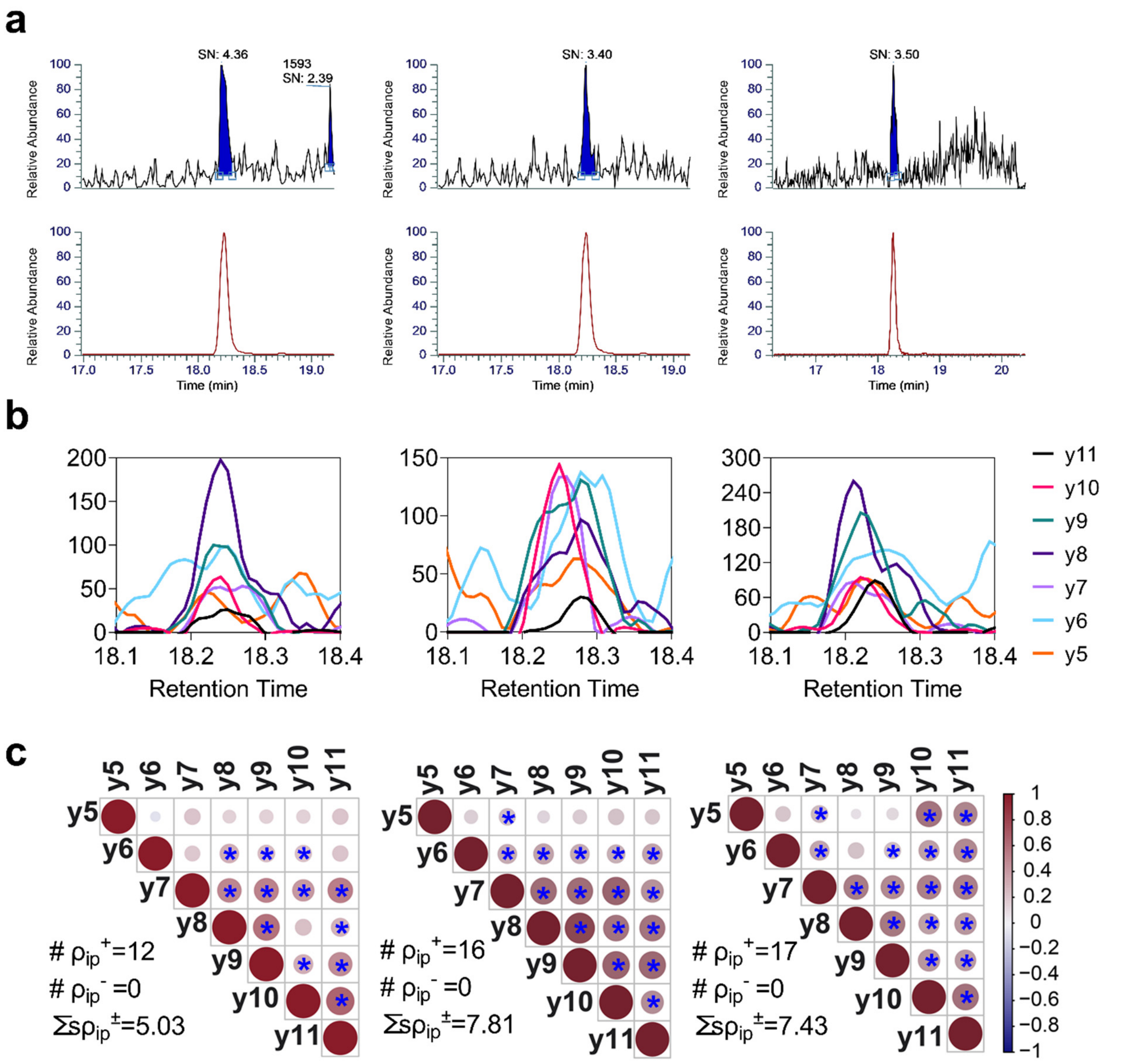

Figure S5. **Peak recognition and differentiation of noise from signal by transition correlation analysis. a** Extracted ion chromatographs for (upper panel) CFP-10pep and (lower panel) its heavy-isotope-labeled IS peptide in affinity enriched samples from three replicates of trypsin-digested healthy human serum spiked with 0.5 pM recombinant CFP-10 and 12.5 nM of the IS peptide. Recognized peaks (blue) are labeled with their signal-to-noise ratio (SN). **b** Targeted retention time window extracted ion chromatographs s for the seven selected transition ions in the blank serum samples in (**a**). **c** Kendall rank order correlation coefficient matrix for the seven

transitions in blank serum samples. Correlation coefficients are color coded, and the circle size represented for their significance p value. Asterisks indicate the correlation coefficients that are significantly different from zero ($p<0.05$). The number of positive and negative correlated transition ions ($\# \rho_{ip}^{+}$, $\# \rho_{ip}^{-}$), and the sum of all significant coefficients ($\sum_{s}\rho_{ip}^{\pm}$) are indicated.

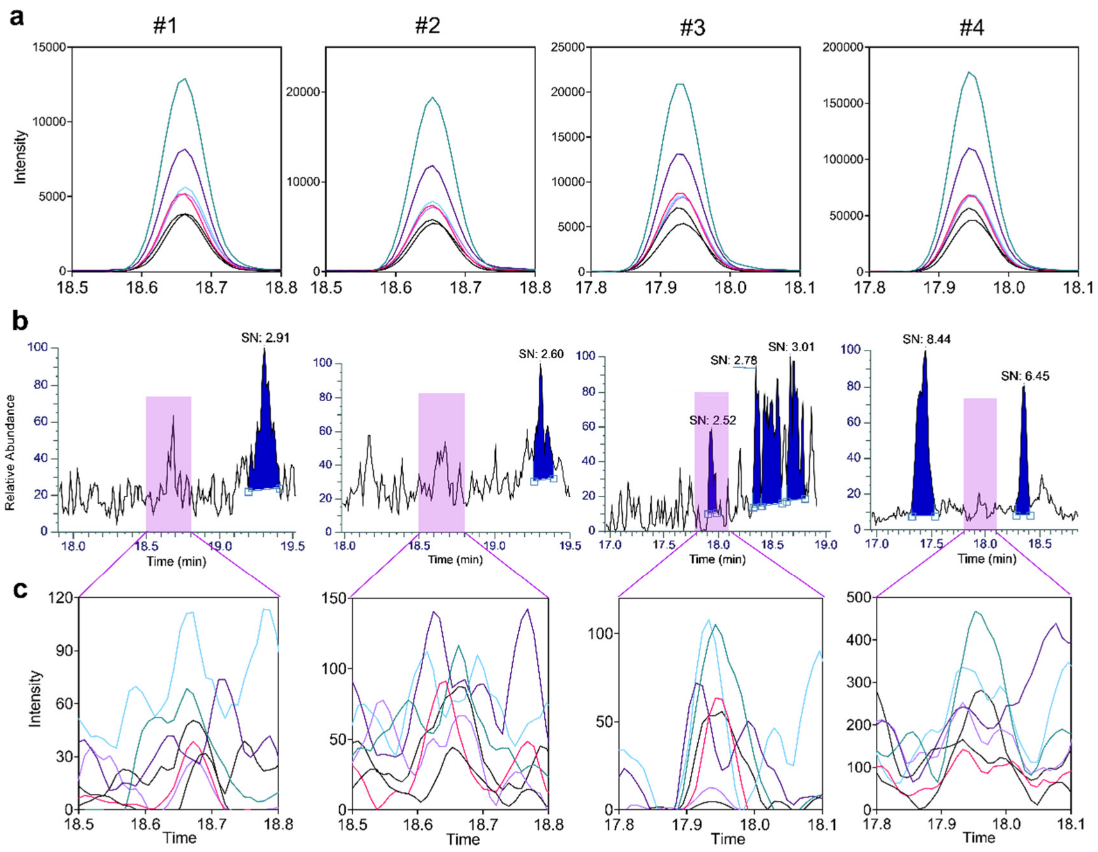

Figure S6. CFP-10pep assay results from four healthy donors with dotp and rdotp values above the selected cutoffs for these parameters (#1-4 in Fig. 2a-b). **a** Extracted ion chromatographs (EIC) peaks corresponding to the integrated target transition ions (y5-y11) of the spiked-in stable-isotope-labeled IS peptide, where elution times for samples 1-2 and 3-4 reflect minor differences in column elution behavior after replacing one column with an identical column. **b** SNRs for non-specific integrated EIC peaks generated by serum-derived transition ions that co-elute with IS peptide transition ions, where blue shading indicates the Genesis algorithm-recognized peaks with SNR values ≥2. **c** EIC peaks for non-specific signals detected within this target window that matched the seven targeted CFP-10pep fragment ions.

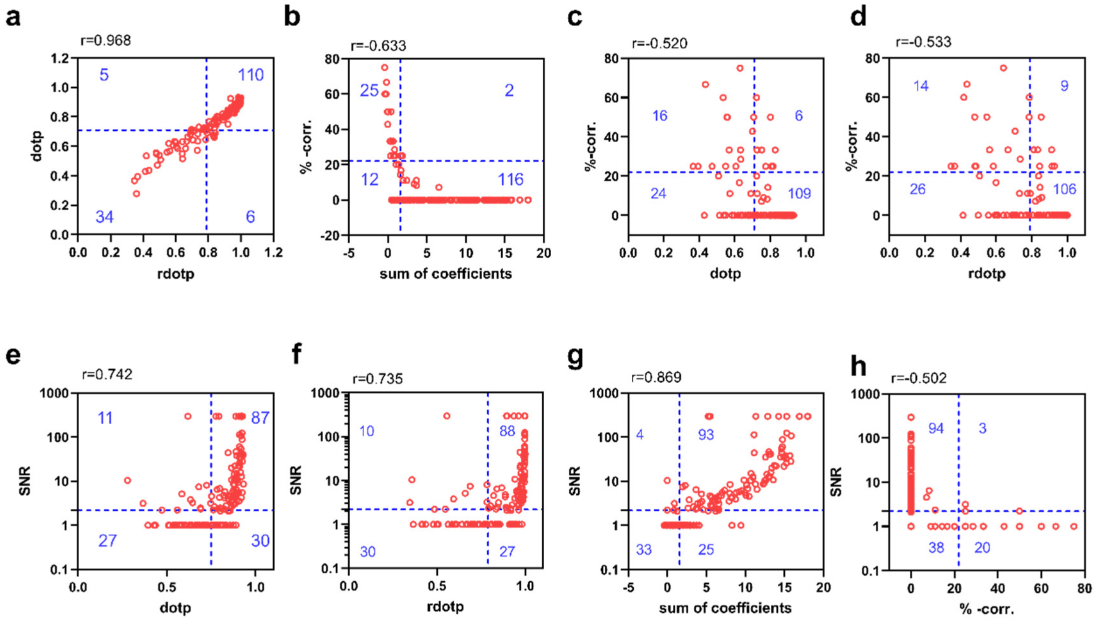

Figure S7. Spearman correlation analysis of peak features. a dotp and rdotp, **b** the percentage of negatively correlated pairs and the sum of Kendall's rank-order correlation coefficients that are significantly different from zero, **c** the percentage of negatively correlated pairs and dotp, **d** the percentage of negatively correlated pairs and rdotp, **e** SNR and dotp **f** the sum of Kendall's rank-order correlation coefficients and dotp, **g** SNR and the sum of Kendall's rank-order correlation coefficients, **h** SNR and the percentage of negatively correlated pairs in the 155 MRM data from the adult TB study cohort shown in **Table 1**.

Table S1. CFP-10pep peak features in triplicates of serum spiked with 0.5 pM CFP-10 or PBS.

	**dotp**	**rdotp**	**SNR**	**ρ_{ip}^{+}**	**ρ_{ip}^{-}**	**$\%\rho_{ip}^{-}$**	**$\sum s\rho_{ip}^{\pm}$**	**Peak area ratio ($x10^{-3}$)**
0.5 pM_1	0.780	0.878	3.400	12	0	0	5.03	1.32
0.5 pM_2	0.835	0.966	3.500	16	0	0	7.81	1.86
0.5 pM_3	0.845	0.942	4.360	17	0	0	7.43	2.41
Mean	0.820	0.929	3.753					1.86
SD	0.035	0.046	0.528					
Criteria	Mean-3×SD							
Threshold	**0.71**	**0.79**	**2.2**					
	**dotp**	**rdotp**	**SNR**	**ρ_{ip}^{+}**	**ρ_{ip}^{-}**	**$\%\rho_{ip}^{-}$**	**$\sum s\rho_{ip}^{\pm}$**	**Peak area ratio ($x10^{-3}$)**
NEG_1	0.621	0.763	1.000	2	3	0.60	-0.18	0.57
NEG_2	0.586	0.662	2.010	5	2	0.29	0.99	0.93
NEG_3	0.772	0.883	1.000	4	2	0.33	1.28	0.94
Mean						0.41	0.70	0.81
SD						0.17	0.78	
95%CI range						0.19	0.88	
Criteria						LL 95% CI	UL 95% CI	
Threshold						**0.22**	**1.58**	

Table S2. Summary of CFP-10pep criteria detected in 20 healthy control samples.

ID	dotp	rdotp	SNR	$\sum_s\rho_{ip}^{\pm}$	$\%\rho_{ip}^{-}$	ρ_{ip}^{-}	ρ_{ip}^{+}	CFP-10^{+}
**C1**	0.86	0.94	1.00	2.90	0.00	0	11	No
**C2**	0.83	0.93	1.00	1.34	0.00	0	3	No
**C3**	0.75	0.92	2.52	4.07	0.00	0	8	Yes
**C4**	0.81	0.92	1.00	0.70	0.00	0	2	No
**C5**	0.72	0.84	1.00	1.06	0.20	1	4	No
**C6**	0.72	0.77	1.00	2.02	0.00	0	6	No
**C7**	0.39	0.37	1.00	1.40	0.25	2	6	No
**C8**	0.43	0.41	1.00	0.48	0.00	0	1	No
**C9**	0.55	0.48	1.00	0.94	0.25	1	3	No
**C10**	0.52	0.64	1.00	0.81	0.00	0	2	No
**C11**	0.37	0.35	3.15	0.91	0.25	1	3	No
**C12**	0.73	0.74	1.00	9.41	0.00	0	20	No
**C13**	0.58	0.59	1.00	0.00	0.67	2	1	No
**C14**	0.54	0.66	1.00	0.00	0.33	1	2	No
**C15**	0.57	0.62	1.00	-0.58	1.00	1	0	No
**C16**	0.66	0.62	1.00	0.10	0.50	1	1	No
**C17**	0.53	0.53	1.00	-1.05	0.71	5	2	No
**C18**	0.68	0.67	1.00	-0.70	1.00	2	0	No
**C19**	0.67	0.68	1.00	-0.25	0.67	2	1	No
**C20**	0.51	0.53	1.00	0.45	0.25	1	3	No

Note: all values that meet the threshold for acceptance are indicated by red text.

Table S3. Estimate of the limit of detection of the CFP-10pep MRM assay.

Replicate	dotp	rdotp	CFP10pep peak area (light)	IS peptide peak area (heavy)	CFP-10pep peak area ratio (light/heavy)
1	0.23	0.33	137	31565.5	0.0043
2	0.25	0.33	344	57385.1	0.0060
3	0.30	0.38	64	47139.1	0.0014
4	0.40	0.41	287	37966.5	0.0076
5	0.57	0.69	106	54459.2	0.0019
6	0.28	0.30	165	128364.7	0.0013
7	0.30	0.38	610	43996.4	0.0139
8	0.61	0.64	514	99722.6	0.0052
9	0.23	0.32	9	18195.9	0.0005
10	0.38	0.40	663	51118.3	0.0130

average CFP-10pep peak area ratio	0.0055
standard deviation	0.0045
limit of detection ratio=average ratio+3*standard deviation	0.0191
limit of detection ratio, log transformed	-1.7188
limit of detection concentration, log transformed	-0.27
limit of detection concentration (pM)	**0.53**
